# Novel insights into the interplay between ventral neck muscles in individuals with whiplash-associated disorders

**DOI:** 10.1038/srep15289

**Published:** 2015-10-16

**Authors:** Gunnel Peterson, David Nilsson, Johan Trygg, Deborah Falla, Åsa Dedering, Thorne Wallman, Anneli Peolsson

**Affiliations:** 1 Centre for Clinical Research Sörmland, Uppsala University, Eskilstuna, Sweden; 2 Department of Medical and Health Sciences, Division of Physiotherapy, Faculty of Health Sciences, Linköping University, Linköping, Sweden; 3 Computational Life Science Cluster (CLiC), Department of Chemistry, Umeå University, Sweden; 4 Institute of Neurorehabilitation Systems, Bernstein Focus Neurotechnology (BFNT) Göttingen, Bernstein Center for Computational Neuroscience, University Medical Center Göttingen, Georg-August University, Göttingen, Germany; 5 Pain Clinic, Center for Anesthesiology, Emergency and Intensive Care Medicine, University Hospital Göttingen, Göttingen, Germany; 6 Department of Neurobiology, Care Sciences and Society, Division of Physiotherapy, Karolinska Institutet; 7 Department of Physical Therapy, Karolinska University Hospital, Sweden; 8 Uppsala University, Public Health & Caring Sciences, Family Medicine & Preventive Medicine Section, Uppsala, Sweden

## Abstract

Chronic whiplash-associated disorder (WAD) is common after whiplash injury, with considerable personal, social, and economic burden. Despite decades of research, factors responsible for continuing pain and disability are largely unknown, and diagnostic tools are lacking. Here, we report a novel model of mechanical ventral neck muscle function recorded from non-invasive, real-time, ultrasound measurements. We calculated the deformation area and deformation rate in 23 individuals with persistent WAD and compared them to 23 sex- and age-matched controls. Multivariate statistics were used to analyse interactions between ventral neck muscles, revealing different interplay between muscles in individuals with WAD and healthy controls. Although the cause and effect relation cannot be established from this data, for the first time, we reveal a novel method capable of detecting different neck muscle interplay in people with WAD. This non-invasive method stands to make a major breakthrough in the assessment and diagnosis of people following a whiplash trauma.

Acute whiplash injury has an annual incidence of 200–300 per 100000^1^,^2^. Whiplash is defined as a sudden acceleration-deceleration movement of the head^3^ transferring energy to the neck, which may result in damage to the soft tissues, nerves and musculoskeletal structures of the neck. The term whiplash-associated disorder (WAD) describes the clinical symptoms related to the injury, and approximately 50% of those affected report persistent disability more than one year after the injury^4^, resulting in both substantial personal and societal costs^1^,^5^. Chronic WAD still remains challenging to treat, and the underlying mechanisms are not well understood due to the limited diagnostic capability of using X-ray or magnetic resonance imaging (MRI).

The cervical spine is supported by the surrounding musculature especially the deep muscle layers which have a unique capacity to contribute to control of intersegmental motion by virtue of their attachments to the cervical spine^6^,^7^. Muscular support maintains postural control of the neck and contributes to eye- and arm-related motor control^8^. Impaired neuromuscular control of the cervical spine has been documented in people with neck pain^9^,^10^,^11^ and an association between neck pain and ventral neck muscle dysfunction has been shown after a whiplash injury and in persistent neck pain^12^,^13^. These findings include reduced activation of the deeper ventral muscles (longus colli and longus capitis)^12^,^13^ and a high prevalence of increased activation of the sternocleidomastoid muscle^13^.

However, no gold standard is currently available for quantifying function of the deep neck muscles. Fine wire electromyography (EMG) of the deep ventral muscles presents risks due to the close proximity of the trachea, oesophagus, major vessels and lymphatic tissue. An alternative EMG approach involving a nasopharyngeal electrode was proposed^13^ and utilised^12^ to measure longus colli and longus capitis activity in people with neck pain however this is also an invasive approach and cannot be applied in routine practice. Moreover, this approach could not distinguish the activity between these two muscles. Functional MRI (fMRI)^14^, MRI^15^ and still image ultrasonography^16^ can be used to visualise the deep neck muscles, but they cannot be used to study muscles in real-time and practically, fMRI is too expensive. However, recent progress in ultrasound imaging and analysis provide the possibility of investigating human mechanical musculoskeletal function^17^,^18^ in real time, *in vivo*, and during functional activity, and can be used to develop new methods that improve diagnostics in WAD.

Skeletal muscles actively contract and produce force in response to control signals from the central nervous system, leading to mechanical changes in the muscles. Ultrasound enables quantitative descriptions of these mechanical changes and allows non-invasive investigation of different muscle layers in real time and *in vivo*^19^,^20^,^21^,^22^. Ultrasound with post-process speckle tracking analysis^23^ measures deformation (mechanical muscle changes, such as elongations and shortenings of the muscle) and deformation rate (how fast the deformation occurs) simultaneously in the superficial and deep neck muscle layers. The interplay between the deep and superficial neck muscles needs to be investigated to improve our understanding of the complexity of neck muscle function^8^ and change in the presence of pain, but challenges in analysing and understanding many highly correlated variables from relatively few unique individuals have caused difficulties in research. However, by using multivariate statistics^24^, it is possible to identify and describe patterns in large data sets^25^, and this method has been used in clinical diagnostic research^26^. Principal component analysis (PCA) and projections to latent structures (PLS)^24^ can be used to identify patterns in multivariate data sets^25^. PCA of real-time ultrasound measurements allows the development of models from complex neck muscle function data. PCA enables the extraction of meaningful information from a large number of variables into relatively few components, and PLS is a regression extension of PCA of variables for underlying correlations and patterns^24^. Partial least squares discriminant analysis (PLS-DA) is a special case of PLS, which can distinguish two groups from each other.

We recently reported that the deformation and deformation rate in three ventral neck muscles, the longus capitis (Lcap), longus colli (Lco) and sternocleidomastoid (SCM), in asymptomatic individuals reveal an individual linear relationship between muscles, but the relationship was weakened or missing in individuals with WAD^27^. However, the complexity of the interaction of the three muscles in total deformation elongation, shortening, and deformation rate could not be investigated with the applied statistical method. Here, we show for the first time that real-time ultrasonography analysed with multivariate statistics can develop a model for mechanical neck muscle function, a model that distinguishes individuals suffering from WAD and healthy controls. Moreover, our findings suggest that the interplay between the ventral neck muscles is less variable in individuals with WAD. The results are promising for improved diagnostics in WAD and for monitoring the response to interventions.

## Results

### PCA of interactions between the three ventral neck muscles

**Figure 1:**
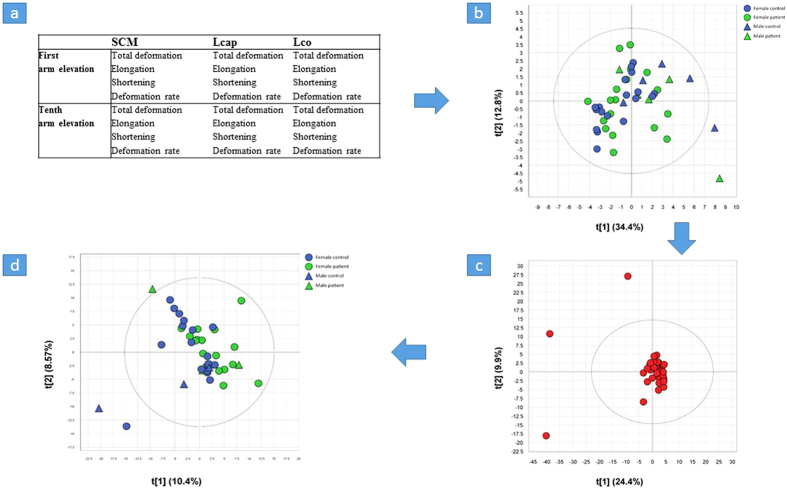
Principal Component Analysis (PCA), scheme of the analysis. (**a**) The 24 variables included in the study were deformation area (% deformation) and deformation rate (% deformation/s) from the Sternocleidomastoid (SCM), Longus capitis (Lcap) and Longus colli (Lco) throughout the first and tenth arm elevation; the area of total muscle deformation; the areas of shortening and elongation deformation; and deformation rate. (**b**) PCA analysis of the 24 original variables, each dot corresponding to one person in the plot, green for patients with whiplash-associated disorder (WAD), blue for controls. The first component explaining 34.4%, the second component 12.8% of the variation in deformation and deformation rate in three ventral neck muscles (SCM, Lcap, Lco) during arm elevation. (**c**) In total, 300 interaction terms were generated for every possible combination of two out of three variable and these can be divided into three different types; 1) interactions in the first arm elevation, 2) interactions in the tenth arm elevation, 3) interactions between the first and tenth arm elevations. The third type is not an actual muscle interaction, but can instead be viewed as a relation between the first and tenth elevation. 24 of the 300 calculated interaction terms were interactions between the same variable, i.e. quadratic terms. This PCA analysis including all 324 variables of deformation and deformation rate. The first two components explained 24.4% and 9.9% respectively and detected three serious (one WAD and two healthy controls) and one moderate outliers, all of them were men. (**d**) PCA analysis with the three serious outliers removed. The score plot showed a more homogenous sample, even if there are some moderate outliers. A small shift to the right in the first component was seen for individuals with whiplash-associated disorder (green dots) compared to healthy controls (blue dots).

A total of 24 variables were included in the PCA: total deformation area, elongation and shortening area, and deformation rate from the SCM, Lcap, and Lco during the first and tenth repetition of an arm elevation task (Fig. 1a). The 24 variables of the original muscle deformations were compressed with PCA into a model comprising five components (R^2^X = 0.73). The first two components, explaining 34.4% and 12.8% of the variation in deformation and deformation rate, were visualized in a score scatter plot (Fig. 1b). This plot can be seen as an overview of the entire sample with each dot corresponding to one person. No difference was found between the patients with WAD (green) and healthy controls (blue) for the first two components (Fig. 1b). Males (triangular dots) tended to have a larger spread than women, and they were also located mostly on the right side of the plot, indicating that men and women have different muscle characteristics. No other components exhibited dis-similarities between the WAD group and control group. The original 24 variables were augmented with all possible interaction terms, including squares, for the 24 variables. The augmented data set, comprising 324 variables, was investigated in an additional PCA model with five principal components (R^2^X = 0.61) (Fig. 1c). The score plot for the first two components explaining 24.4% and 9.9% of the variation in deformation and deformation rate, revealed three serious outliers and one moderate outlier, all of them men. These individuals showed a deviating pattern in neck muscle interaction. The corresponding loading plot (data not shown) did not show any anomalous variables, or muscle interactions that could explain the behaviour of the outliers. The score plot for the updated PCA model (Fig. 1d) with the three outliers removed showed a more homogeneous pattern, though with some moderate outliers. The plot also shows that individuals with WAD are positioned to the right to a higher degree, which indicates there is difference between the WAD and control groups.

### PLS-DA including 324 variables

**Figure 2:**
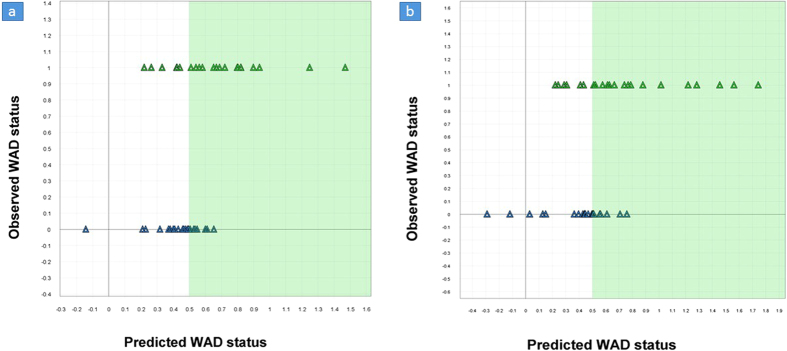
Partial least squares discriminant analysis (PLS-DA). (a) PLS-DA including all 324 variables enable class separation (discrimination) analysis, in this case between individuals with whiplash-associated disorders (WAD) and healthy controls. The upper line (green triangle) shows the WAD group with a significant greater spread compared to the controls (lower line, blue tringle) and 72.1% of the individuals were correctly classified. The shaded area represents the 0.5 discriminant cut-off. (**b**) PLS-DA including the 80 most important variables. The class separation (discrimination) analysis between individuals with WAD (green triangles) and healthy controls (blue triangles) showed a model with higher prediction efficiency (R^2^Y = 0.72, Q^2^Y = 0.59) than PLS-DA with 324 variables (R^2^**Y **= 0.50, Q^2^**Y **= 0.21). However, the observed predictive outcome were similar for the two models. The shaded area represents the 0.5 discriminant cut-off.

A one-component PLS-DA model (R^2^**Y **= 0.50, Q^2^**Y **= 0.21) was created in order to span the co-variation between **X**, comprising all 324 variables, and with the patient status (WAD patient or control) as a single **y** variable. Accordingly, this model reveals differences between the WAD and control groups in terms of the involved muscle interactions. As given by R^2^**Y**, the model indicates that an evident connection exists between the muscle interaction data and patient status. As given by Q^2^**Y**, the prediction efficiency shows a weaker but adequate model for this purpose. To further validate the methodology and prediction efficiency, a procedure involving test sets was employed. The procedure excluded one-third (14–15) of the individuals from the creation of a reduced PLS-DA model (comprising 28–29 individuals) of the co-variation between the 324 variables and the **y** variable (WAD patient or control). The patient status (WAD or control) was then predicted for the excluded one-third (14–15 individuals) by the reduced PLS-DA model. The procedure was repeated two additional times, followed by consolidation of the prediction outcome of all three procedure runs. The observed versus predicted plot (Fig. 2a), showing true observed values plotted against the predicted outcome, indicated a smaller spread for the control group (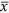 = 0.43, s = 0.17) compared to the WAD group (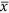 = 0.67, s = 0.30). Using the control individuals as population I and patients with WAD as population II, the zero hypothesis of equal sample means was rejected at alpha = 0.01 (p = 0.017) and the two-sample t-test assuming unequal variances was used (p = 0.0022). Normal distribution of data was assumed, and the two populations were assumed to have originated from random sample data. Furthermore, using a discriminant cut-off of 0.5, a total of 12 individuals were wrongly classified as WAD (seven healthy controls) or controls (five WAD). Thirty-one (72.1%) of the individuals were correctly classified. Though not presenting an ideal prediction outcome, the validation proves the connection between the muscle interaction data and WAD patient status.

### PLS-D analysis and variable influence of projection (VIP)

**Figure 3:**
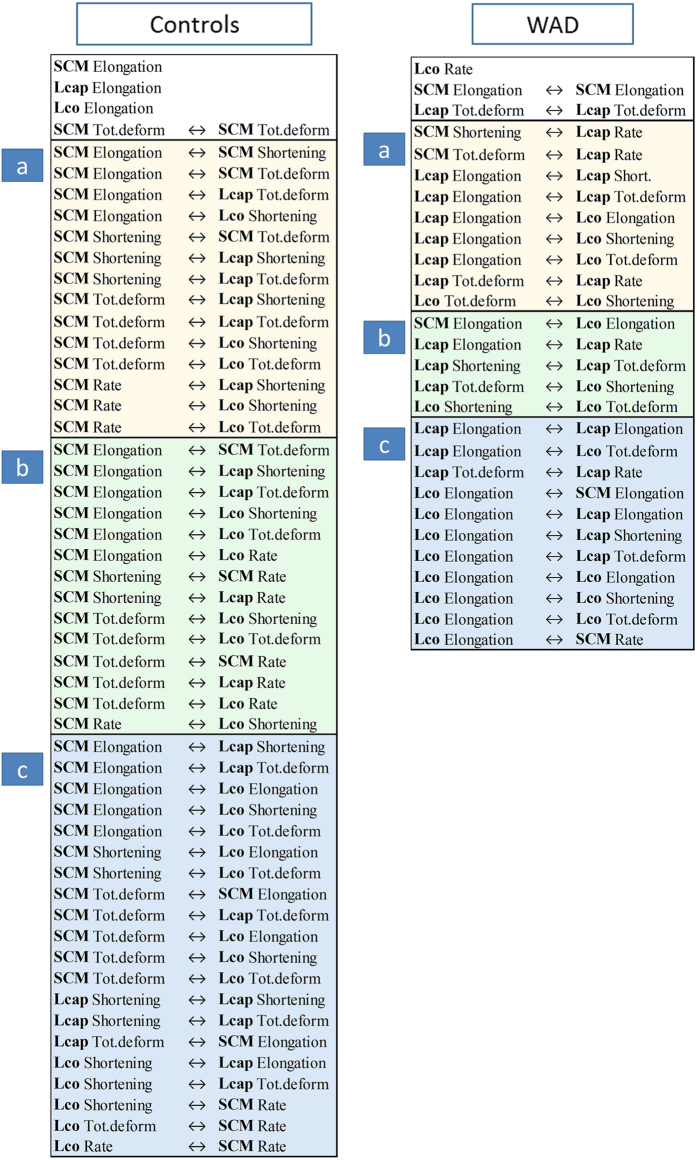
Variable influence on projection (VIP). To determine the most important variables for interaction between deformation and deformation rate, variable influence of projection (VIP) was used and 80 variables were detected. The three ventral neck muscles are; Sternocleidomastoid (SCM), Longus capitis (Lcap), Longus colli (Lco). The majority (52) of the variables, were positively correlated to healthy controls. The remaining 28 variables were related to muscle interactions or muscle relationships in the WAD group. The white fields are quadratic interactions. A quadratic interaction term describes the interaction between a variable and itself. Quadratic term may be added to model non-linear effects. (**a,b)** Two-way interactions between neck muscles in deformation and deformation rate during the first (yellow fields) and tenth arm (green fields) elevation, showing the most prominent loadings that separate WAD from controls. In controls, 14 interactions were seen during the first and tenth arm elevation. For the WAD group, 9 interactions were present for the first arm elevation which decreased to 5 in the tenth arm elevation. The total deformation (Tot.deform.) denotes the sum of shortening and elongation (deformation %); shortening represents muscle shortening and elongation represents muscle elongation (deformation %); rate denotes the deformation rate (deformation %/s) in the muscle during one arm elevation. (**c)** The blue fields are interaction terms between the first and tenth arm elevation but are not indicative of actual muscle interplay. The interaction should be seen as a relationship between the first and tenth arm elevation that distinguishes the WAD and control group in this multivariate model. Relations in neck muscle deformation and deformation rate between the first and tenth arm elevation, showing the most prominent loadings that separate the two groups. Twenty interactions in the control group and 11 in WAD. Abbreviation: SCM, sternocleidomastoid; Lcap, longus capitis; Lco, longus colli.

In order to analyse the content of the PLS-DA and, more specifically, the included muscle interactions, all 324 variables were ranked according to their variable importance or VIP^24^. To interpret a PLS model with many variables is a complex task and the most important variables can be detected using VIP. The higher the VIP value (with cut-off value often 1.0 or higher), the more influence the variable has on the explained **y** variable. The effective cut-off value in the present study was 0.99, which corresponded to the 80^th^ variable. VIP sorting made it possible to summarize the variables, or in this case muscle interactions, primarily involved in the difference in muscle interaction between WAD patients and healthy controls. Accordingly, 80 variables exhibiting the highest VIP values were selected for further analysis (Fig. 3). A two-component PLS model (R^2^Y = 0.72, Q^2^Y = 0.59) was calculated to describe the relationship between **X** and **y** while simultaneously verifying the selection of the most important variables. Although the VIP ranking enabled a model with what would seem to be a high prediction efficiency, the observed prediction outcome using an identical validation procedure as the full model was similar to the previous model. Twenty-nine of 43 individuals were classified correctly using the two-sample t-test assuming unequal variances (p = 0.0014; see Fig. 2b). The data comprises a set of correlated variables with a high redundancy of information that co-varies with **y**. The 80 most prominent variables were able to retain most of the prowess of the original model (Fig. 3).

**Figure 4:**
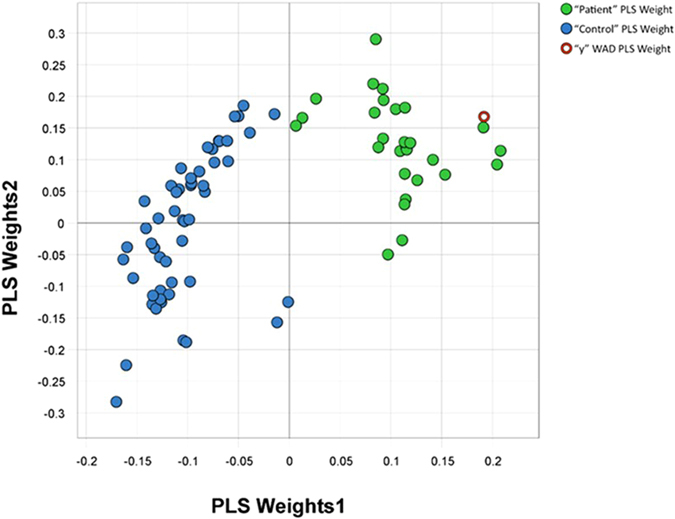
Projection to latent structure (PLS) weights in individuals with whiplash-associated disorder (WAD) and healthy controls. PLS is a regression extension of principal component analysis and revealed that 52 interactions (variables) between deformation and deformation rate in the three ventral neck muscles were positively correlated to healthy controls (blue dots). The remaining 28 variables, of the in total 80 variables, were correlated to WAD (green dots).

A PLS weight plot was created to explore the characteristics of the 80 most important interactions (Fig. 4). The weight plot reveals that a majority (52 variables) positively correlated with healthy controls. The remaining 28 variables were directly related to muscle interactions or muscle relationships in the WAD group. A summary of the interaction terms is provided in Fig. 3.

### Fewer interactions between neck muscles for the WAD group

The WAD group had fewer interactions between muscles compared to the control group during the first arm elevation (WAD: 9 (39%), controls: 14 (61%); Fig. 3a). The SCM was involved in all interactions in the control group (SCM 100%, Lcap 43%, Lco 35%). Lcap was part of most of the interactions during the first arm elevation in WAD (SCM 22%, Lcap 89%, Lco 44%). The interactions between muscles included elongation for the two deepest muscles in the WAD group, Lcap and Lco, which was not seen in controls, possibly indicating less stability of the cervical spine in WAD.

The WAD group had decreased interactions between muscles at the tenth arm elevation compared to the first elevation, and fewer interactions than controls (WAD: 5 (26%), controls: 14 (73%); Fig. 3b). SCM was only present in one interaction in the WAD group (SCM 20%, Lcap 60%, Lco 60%), and in the control group SCM was involved in all 14 interactions (SCM 100%, Lcap 28%, Lco 50%).

The deformation rate in the Lcap interacted with muscle deformation in the same muscle and the SCM in the patients with WAD. In controls, deformation and deformation rate interactions were observed between SCM/Lcap and SCM/Lco during the first arm elevation. Interactions between deformation in SCM and deformation rate in all three neck muscles during the tenth arm elevation distinguished the controls from the WAD group (Fig. 3b). The third interaction type is not an actual muscle interaction, but can be viewed instead as a relationship between the first and tenth arm elevation, distinguishing individuals with WAD and healthy controls (Fig. 3c). Fewer interactions were observed in the WAD group (11 (35%)) compared to controls (20 (65%)). In the WAD group, 91% of the interactions included elongation of the deep neck muscles (Lcap and Lco), and SCM was not presented during the first arm elevation. For controls, SCM was involved in the interactions at the first arm elevation and the feature of the deep muscles was shortening.

## Discussion

### Comparisons between WAD and healthy controls

The use of multivariate techniques, in this case PCA and PLS, provided a fully viable analysis of deformation and the deformation rate and their interactions in individuals with chronic WAD and in pain-free individuals. The data comprising a set of 324 highly correlated variables was compressed to a one-component PLS-DA model that revealed the differences between the WAD group and healthy controls. The relationship found between the deformation and deformation rate data and patient status was significant, although it was associated with a moderate Q^2^**Y** value of 0.21. For PLS-DA modelling in general, a Q^2^**Y** of 0.5 can often represent a nearly ideal prediction outcome for more heterogeneous samples. Therefore, values close to unity are not attainable in general and are limited to the modelling of extremely homogeneous samples. The use of the 80 most prominent variables, as given by the VIP ranking, resulted in a model with a high Q^2^**Y** value, indicating a high prediction efficiency (Q^2^Y = 0.59). The observed prediction outcome for the reduced model, 29 out of 43 individuals, was similar to that of the full model where 31 out of 43 individuals were correctly classified. This finding confirms the ability of the reduced model to almost fully resolve the full model and illustrates the high redundancy of the initial data.

### Validation of the model

The rationale behind using the test set validation, where one third of the individuals were left out and predicted at a time, was to create a more robust validation procedure for the sample data, comprising 43 individuals in total. In multivariate modelling in general, cross-validation routines normally employ a more conservative exclusion scheme where only a few individuals are left out for prediction at a time. This allows for only small deviations from the full model, and basically means a similar model is used for all predictions. Excluding a larger portion of the sample data will on the other hand allow for a larger deviation from the original model. This implies that a more independent model can be created for each validation round and more importantly, the final prediction outcome will be less dependent on more or less the same model. The validation was primarily undertaken to ensure the data contained variation that actually reflected differences between individuals with WAD and healthy controls. In fact, the observed predicted classification can be translated to a highly significant difference between the two groups. Nevertheless, from a classification point-of-view, the results were not optimal. However, there is likely several other factors which contribute to the variation between individuals with persistent disability after a whiplash injury and healthy controls including post-injury psychological factors such as fear of movement^4^.

The cross-validation procedure gave a seemingly high Q^2^Y value as all observations/individuals (including those that were to be predicted) were used for deriving the 80 most prominent variables. In other words, the observations to be predicted were influencing the VIP selection and hence they were not fully unknown to the model. This was a limitation by running the software in default automatic mode. However, the test set procedure, performed manually, avoided this problem by excluding the observations to be predicted before the VIP selection was derived. As such, they were completely unknown to the model.

For interpretation purposes, the reduction to 80 variables enabled a straight-forward characterization of primary muscle interactions as either WAD or controls. Accordingly, two separate muscle interaction models could be derived for the two groups. Men in both groups (WAD and controls) had a larger spread in interactions between muscles than women. Men and women have different muscle characteristics with larger muscle size and greater strength for men^28^, which may explain the greater variability in interactions for men in this study.

### Mechanical neck muscle behaviour and measurement methods

In the present study, involuntary neck muscle mechanical behaviour, including muscular control of neck posture, was investigated during voluntary arm elevations. Pain is known to affect muscle movement^29^,^30^,^31^,^32^,^33^,^34^,^35^ and muscle synergies^36^,^37^ resulting in altered neck muscle function^38^. Moreover, there may be changes in physiological involuntary variables in the central nervous system^39^ for example reflex mediated eye movement and postural control^40^,^41^. To some extent, the current results are different from earlier research^14^,^42^,^43^ that reported decreased and delayed muscle activation in the deep ventral neck muscles and increased activity in the superficial SCM^13^,^31^,^44^,^45^ in individuals with chronic neck pain and WAD. However this could be attributed to the use of different measurement methods and the variables being measured. A feature of skeletal muscles is the possibility to activate in different ways under differing force/load. Concentric contraction implies active shortening of the muscle, eccentric contraction involves active elongation, and isometric contraction is when the muscle actively holds a fixed length. The muscles can also passively stretch (elongate) or have low activity at rest. Real-time ultrasound with speckle tracking measures the dynamic change in the muscle length (shortening and elongation), and can be passive or caused by a concentric or an eccentric contraction. The different measurement methods can explain some of the discrepancies between the current results and those from EMG and fMRI studies. EMG captures nerve and muscle interactions and fMRI measures the enhancement of the T2 relaxation time of muscle water after activity. Notwithstanding, it is relevant to highlight that calculating the deformation area and deformation rate and analysing the muscle interaction with multivariate statistics as in the current study, revealed separate muscle models for WAD and controls.

### Separate muscle interaction models for WAD and controls

In the current study, individuals in the WAD group showed interactions in the deep neck muscles that separated them from healthy controls. Stabilization of the cervical spine is considered to be maintained by the deep neck muscle layers^6^,^7^ and the deep neck muscles (Lcap and Lco) were part of most of the interactions in the WAD group. However, many of the interactions involved elongation of the deepest muscles suggesting that stabilization of the cervical spine did not occur, but shortening of these muscles was also seen. The deformation values must be interpreted with caution as deformation is not necessarily a direct measure of muscle activation. Deformation can occur due to pressure from nearby tissues or by passive elongation or pressure in parts of the muscles not shown in the ultrasound image.

Changes in head kinematics could have potentially affected the deformation measures and any conclusion on cause or effect cannot be drawn. However, we measured the ventral neck muscles during arm elevation to 90 degrees and the subjects were specifically instructed to hold their head steady during the test. Thus, we believe that it is reasonable to assume that head movement has not affected the data and does not significantly account for the difference between groups.

The postural test of the neck muscles in the present study showed an important difference between WAD and controls, with interactions including more shortening for the deep muscles in healthy controls. It could be speculated that shortening reflects an active muscle contraction since few other tissues would be expected to induce pressure on the longus capitis and longus colli, however this cannot be confirmed from the current data. Moreover, elongation of the deep muscles in the WAD group may reflect passive elongation and difficulties to maintain postural control. The WAD group had fewer interactions between muscles compared to controls and the superficial muscle (i.e., SCM) was only involved a few times. It should be noted that movements generally involve components that are task directed and components that are postural^46^, and pain could impact on the task-directed or postural component, or both^30^. In this study we largely evaluate the postural role of the deep ventral neck muscles during an arm elevation task which contrasts to some earlier work evaluating deep ventral neck muscle activity during voluntary contractions^42^. The superficial muscle, SCM, was involved in all fourteen interactions in the control group during both the first and tenth arm elevation, for the WAD group only in two interactions during the first arm elevation and one during the tenth. This is different from earlier studies that reported increased activity in the SCM after experimentally induced neck pain^29^ and in individuals with chronic neck pain compared to healthy controls^13^,^31^,^44^,^45^ when measured with EMG or fMRI. But again, mechanical measures of muscle function as performed in the current study are not synonymous with measures of muscle activation.

### Variability in the interplay between different muscles

Motor function is inherently variable. There are multiple ways to achieve a goal involving different combinations of muscle activity, different coordination between body segments, and multiple possible control strategies. Variability can be helpful as variation in movement “shares” the load around structures so that one tissue or structure is not repeatedly loaded^36^. The greater number of interactions in the control group could potentially be seen as an indicator of greater variability in the interplay between neck muscles. This may be due to a flexible solution in asymptomatic individuals when several muscles are available to control the cervical spine and perform arm elevations relying on the redundancy of the neck muscles. Low variability in the WAD group may suggest that the task was performed using fewer muscles, in a more stereotype manner. During the tenth arm elevation, the interactions between muscles decreased for the WAD group, showing interactions mostly between the two deep neck muscles, Lcap and Lco. In the control group, 14 interactions were observed during first and tenth arm elevation, and the SCM was involved in all interactions, showing higher variability in muscle interactions to maintain postural control of the neck when voluntary arm elevation continued. Recent work^34^ shows that some people use the same muscle synergies during multi-joint planar reaching tasks in non-painful and painful conditions, which is consistent with the observation that some people perform a particular task in a more stereotyped manner than others^33^. Those individuals with less variable motor programs seem to be those more prone to develop pain as they overuse the same strategy rather than taking advantage of the redundancy of the motor system^33^. Provocation of neck pain during activity^35^ may also occur if load sharing between muscles is reduced. In the present study, the WAD group displayed a further reduction in the number of muscle interactions as the activity progressed, which could negatively affect their work capacity. Lack of variability of muscle use may predispose to higher fatigue and pain^47^ and decreased motor variability during repetitive work has been reported in people with chronic neck-shoulder pain^32^. The relatively few muscle interactions in the WAD group could also be attributed to fear-avoidance^48^. If pain is seen as a threat of existing or impending neck injury, then it can lead to avoidance of movement and potentially reduced variability of neck muscle use.

Our innovative results revealed two different neck muscle interaction models in individuals with chronic WAD and healthy controls. Moreover, we detected less interactions and fewer neck muscles involved in the two-way interaction in chronic WAD, potentially indicating less variability in the use of ventral neck muscles for individuals with WAD. The predictive value of the most important 80 muscle interactions was relatively high, but it is likely that neck extensor muscle dysfunction may also contribute to WAD and further studies of the neck extensor muscles are required. The methods in the present study show great promise for improved diagnostics in WAD.

## Methods

### Participants

Data from ultrasound imaging^27^ was analysed using multivariate statistics to develop a new model for ventral neck muscle interaction.

Twenty-three individuals, 18 women and 5 men (mean age 36 years; SD 11.2), with persistent WAD (mean time since whiplash injury 22 months; SD 7.7), an average pain intensity over the last week of 51 mm (SD 17.6) on the visual analogue scale (VAS; 0 = no pain, 100 = worst imaginable pain), and neck disability rated as 34% (SD 13.8) on the Neck Disability Index (NDI; 0% = no disability, 100% = highest score for disability) were recruited consecutively for ultrasound investigation from a randomized controlled trial^49^. The WAD group was compared to 23 healthy controls matched for age and sex (mean age 36 years; SD 10.9) with a pain intensity of 0.7 mm (SD 1.0) and NDI 1% (SD 1.6).

Study inclusion criteria for the randomized controlled trial were persistent symptoms associated with a whiplash injury 6 months to 3 years prior to study entry; WAD grade II (neck pain and musculoskeletal signs) or III (neck pain plus neurological signs); age 18–63 years; and persistent neck pain rated greater than 20 mm on a VAS and/or neck disability greater than 20% measured with the NDI. For eligibility in this ultrasound study, individuals also had to report neck pain on the right side of the neck and right-handedness. Exclusion criteria were signs of traumatic brain injury at the time of whiplash injury; known or suspected serious pathology; previous fracture or luxation in the cervical spine; contraindication to exercise; neuromuscular diseases; rheumatologic disease; previous neck pain causing more than 1 month of sick leave in the year before the whiplash injury; severe mental illness; current alcohol or drug abuse; or an inability to understand spoken and written Swedish.

The healthy controls were recruited from university staff, hospital staff, and acquaintances. Inclusion criteria were no present or past neck problems; no trauma to the neck or head, including whiplash injury; no neck or low back pain; no rheumatologic or neurologic disease; and no generalized myalgia.

The study was approved by the Regional Ethics Review Board, Faculty of Health Science Linköping University and conducted according to the Declaration of Helsinki. Written informed consent was obtained from all participants

### Ultrasound measurement

**Figure 5:**
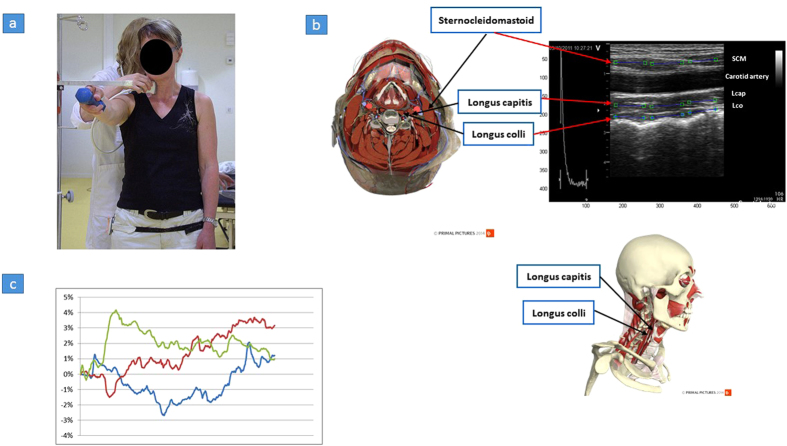
Ultrasound imaging and speckle tracking analysis of ventral neck muscles. (**a**) Ultrasound imaging was recorded during 10 repetitive arm elevations. The arm was raised in flexion to 90 degrees, to an adjustable horizontal bar positioned so that the index finger would touch the bar. Customized contact switches were attached, one on the right wrist and one on the right hip. The contact signals indicated the start and stop of arm movements which were recorded by the ultrasound device allowing synchronization of data. To maintain a steady pace during the examination, a metronome was set at 40 beats per minute. The individual was then asked to hold the head steady, to look at the bar, and lift their arm to the bar with the beat and then lower the arm to the switch contact on the next beat. (**b**) The three ventral neck muscles, superficial sternocleidomastoid (SCM) and the two deep muscles longus capitis (Lcap) and longus colli (Lco) are shown in muscles (credit: Primal Pictures Ltd) and ultrasound images. The ultrasound image shows the longitudinal projection with the superficial SCM shown at the top, followed by the common carotid artery, Lcap and Lco. A region of interest (ROI) was manually placed in the first frame of the video sequence of the muscle, measuring the deformation (elongation and shortening) and the deformation rate (how fast the shortening and elongation occurs). Three ROIs (each indicated as a blue line with a square at each end) were placed in each muscle. (**c**) This diagram illustrates muscle deformation sequences during one arm elevation. The three different patterns of muscle deformations area for three different individuals. A line represents the changes observed in the ROI (deformation %) in one muscle, during one arm elevation. Muscle shortening is the region (area) below zero (negative values) and muscle elongation represents the region (area) above zero (positive values). The sum of negative and positive areas represents the total muscle deformation during one arm elevation. When the line crosses 0%, the muscle deformation shifts from shortening to elongation, or vice versa.

The ventral neck muscles were evaluated with a B-mode, 2-D ultrasound Vivid-I scanner (GE Healthcare, Horten, Norway) connected to a hand-held 12 MHz linear transducer (38 mm) with a high frame rate (235 frames/s). SCM, Lcap, and Lco were recorded during 10 repetitive arm elevations, and ultrasound images (“video” sequences) were acquired during the first and tenth arm elevations (Fig. 5a). The first arm elevation was recorded to evaluate the interplay between muscles at the start of the task and the tenth arm elevation was recorded to identify whether a short repetitive arm elevation task (mean time 2.4 seconds) changes the deformation and/or deformation rate in the ventral neck muscles. Each arm elevation included approximately 570 frames and the capacity of the ultrasound device did not allow all ten arm elevations to be recorded. All ultrasound measurements were performed with the transducer held in a longitudinal position at the level of the fourth cervical vertebra (C4) on the right side of the neck. The C4 level was verified with a transverse ultrasound projection of the bifurcation of the carotid artery usually observed at the level of C4. The transducer was then rotated longitudinally and all ultrasound video recordings of the SCM, Lcap, and Lco were performed in this longitudinal position. Each participant was asked to stand in a comfortable upright position with their feet behind a line marked on the floor, holding a weight of 0.5 kg (women) or 1 kg (men) in the right hand. For familiarization with the test, the individual practiced the test with the left arm. Two experienced physiotherapists carried out the ultrasound test, one performed the ultrasound examination and the other assisted the ultrasound examiner and instructed the participants. The experiment was designed to investigate neck muscle function during repeated arm lifting. In our clinical experience, increased pain is commonly reported across arm lifting in patients with chronic WAD, and an activity-related summation of neck pain has been described^35^.

### Speckle tracking

The speckle tracking method was based on an algorithm developed by Kanade-Lukas-Tomasi (KLT)^50^,^51^, which was further enhanced with the methodology described by Farron *et al.*^52^. The speckle tracking methodology was implemented with an in-house software program written in Matlab 2013b^53^. The KLT tracking algorithm was part of the Computer Vision toolbox in Matlab. In the first frame of the video sequence of the muscle, a region of interest (ROI) was manually placed, making it possible to track the unique speckle pattern frame by frame through the video sequence (Fig. 5b). The ultrasound video images were coded during the post-process analysis. Accordingly, the analyser was blinded to group affiliation. The ROI consisted of a large number of measuring points, and the frame to frame displacement could be obtained with a least squares fit assuming a linear strain model. Therefore, as the speckle pattern changes length with muscle activity, so does the length of the ROI. *Muscle deformation* (elongation or shortening) was calculated as the percentage change from the original length of the ROI compared to rest (expressed as % deformation). The *muscle deformation rate* was expressed as the amount of deformation per time unit (% deformation/s). Three ROIs (each 10 × 3.3 mm) were positioned longitudinal to the muscle fibres in each muscle; together, the three ROIs covered 30 mm of the unique speckle pattern in the muscle of interest. To assess muscle deformation, the areas on the deformation curves were calculated (Fig. 5c). The trapezoidal rule (Eq. 1) where A is the area, t is time between samples and y_n_ is the current ROI position at sample point n, was used as basis for the area calculation. To handle intersections with the 0% line, the equation was modified. Linear interpolation was used to estimate additional sample points with adjusted t-values at intersections with the 0% line. Thereby, the area under and the area above the 0% line could be separated.


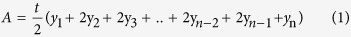


Deformation rate results are presented as the root mean square (RMS), which gives information about the local tissue velocity of deformation. Speckle tracking has been validated, showing a method to assess the contractile performance of cardiac and skeletal muscles^54^ and that muscle deformation measured with speckle-tracking are related to the magnitude of muscle activity and force during progressive electrical stimulation measurements of the biceps brachi muscle^21^. However, changes in head kinematics could have potentially affected the deformation measures in this study and more studies are required to validate this method against force measurements for neck muscle activation. Thus, any conclusion on the degree of active or passive muscle deformation can not be drawn from this study. Nevertheless, the model developed in the present study was able to detect differences between individuals with WAD and healthy controls.The test-retest reliability of the speckle tracking analysis method is excellent (ICC 0.71–0.99)^55^.

### Other measurements

Prior to ultrasound imaging, the participants completed a baseline questionnaire to obtain details of their age, gender, average pain intensity experienced over the prior week (VAS), and NDI. The WAD grade, body mass index (BMI), neck fatigue before and after the test (Borg CR-10 scale: 0 = no fatigue, 10 = extremely strong fatigue)^56^, and activity level (activity index: 1 = inactivity, 2 = low activity, 3 = moderate activity, 4 = high activity)^57^ were also recorded (Table 1).

### Statistical and multivariate data analysis

The variables included in the study were deformation area (% deformation) and deformation rate (% deformation/s) from the SCM, Lcap, and Lco during the first and tenth arm elevation. A total of 24 variables were derived for the three neck muscles (Fig. 1a). Interaction terms were then generated for every possible combination of two out of three variables (Fig. 1b). Prior to calculating the interactions, each variable was mean centred and scaled to unit variance. The interaction between variables *a* and *b* can be described as the element-wise multiplication, where 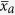 and 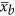 are the means of the two variables *a* and *b* and *s*_*a*_and *s*_*b*_are the standard deviations (Eq. 2), The quadratic terms were logarithmically transformed with base 10 prior to further analysis.


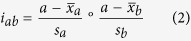


The 300 variables, with the 24 original muscle deformation variables, were assembled as an **X**-matrix and subjected to multivariate data analysis. Auxiliary variables, such as age, gender, pain intensity, neck disability, WAD grade and WAD status (patient or control), BMI, fatigue, and activity level, were also added to the dataset. The auxiliary variables were used as responses, **Y**, or merely for interpretation in which the variable could be used to colour the observations in a plot. The two sample groups were checked for normality using; visual inspection of the distribution showing fairly normal distribution; and the Lillifors test at a significance level of p < 0.05. For both sample groups the null hypothesis could not been rejected (WAD group; p = 0.063 and control group; p = 0.42), thus both groups were normally distributed. The two groups were checked for equal variances with an F-test, which resulted in a rejected null hypothesis (p = 0.017). Therefore, we used the two-sample t-test assuming unequal variances for differences between the WAD and control groups.

Multivariate data analysis using PCA and PLS was performed in Simca 13.0 (MKS Umetrics, Umeå, Sweden) and Evince 2.6 (UmBio AB, Umeå, Sweden). In general terms, multivariate data analysis makes it possible to identify groups of correlated variables. In the present study, the analysis was specifically employed to investigate how three ventral neck muscles are correlated by analysing interaction terms. The strength of the multivariate models in this study was determined by R^2^**X**, the explained variance in the **X**-matrix; R^2^**Y**, the explained variance in the **Y**-matrix; and Q^2^**Y**, the predictive explained variance in **Y**. The maximum value for each is 1.0 and symbolizes a perfect model.

The models were validated with respect to quality and significant components, with leave-out-p cross-validation, where p equalled one-seventh of the observations. Moreover, all Q^2^**Y** values reported in the current work were derived from the cross-validation. The dataset and its models were also validated by using internal test sets. The test set procedure implied the exclusion of a certain number of observations, which were then predicted by the model created from the remaining observations. In the present study, PCA scores were used for an explorative analysis of the data. Overviews of the data, so-called score plots, were used to find groupings and possible outliers. PCA loadings were used for model interpretation (e.g., to investigate differences between the WAD group and healthy controls). PLS was used to build regression models between the variables in **X** and **Y**, such as WAD status. PLS was also used to find the most prominent variables among the interactions, denoted by the VIP.

## Additional Information

**How to cite this article**: Peterson, G. E. *et al.* Novel insights into the interplay between ventral neck muscles in individuals with whiplash-associated disorders. *Sci. Rep.* **5**, 15289; doi: 10.1038/srep15289 (2015).

## Figures and Tables

**Table 1:** Characteristics of participants with whiplash-associated disorders (WAD) and pain-free controls.

	WAD (N=23)	Control (N = 23)	p
WAD grade II/III (number)	16/7	0	<0.001
BMI^a^ male (mean and SD)	24 (6.6)	24 (3.6)	0.95
BMI female (mean and SD)	27 (7.9)	22 (2.3)	0.03
Physical activity level^b^ (median and IQR)	2 (2–3)	4 (3–4)	<0.001
Neck muscle fatigue (median and IQR)^c^	4.0 (2.0–5.0)	0.0 (0.0–0.0)	<0.001

^a^BMI; Body Mass Index (kg/m^2^).

^b^Physical activity level over the prior 12 months (1 = inactivity, 2 = low activity, 3 = moderate activity, 4 = high activity).

^c^Neck muscle fatigue measured on a Borg fatigue scale (0 = no fatigue, 10 = extreme fatigue).
